# G. Dundas Edwards

**Published:** 1905-12

**Authors:** 


G. DUNDAS EDWARDS, M.A., M.R.C.S., L.R.C.P.

WE regret to record the death of Dr. Edwards, who died suddenly on October 1st in London. After taking his arts degree at Cambridge Dr. Edwards became a student at the Bristol Medical School and Royal Infirmary, and afterwards began practice in Bath; but very soon he contracted phthisis, and had to relinquish all work for some years. He subsequently spent some winters in Egypt, finally settling down at Assouan. Here his knowledge of men, tact, continental education, and linguistic abilities speedily ensured him success in the difficult work of medical practice in a health resort. He spent the summers in England, but his phthisis never really healed, and he was always glad to get back to sunnier climes. He will long be remembered by Bristol medicals as a finished and polished actor, and by his friends as a most delightful and amusing companion and raconteur. His little work on the climatic conditions and advantages of Assouan was reviewed in the March number of this year.

